# Morals, morale and motivations in data fabrication: Medical research fieldworkers views and practices in two Sub-Saharan African contexts

**DOI:** 10.1016/j.socscimed.2016.08.019

**Published:** 2016-10

**Authors:** Patricia Kingori, René Gerrets

**Affiliations:** a The Ethox Centre, Nuffield Department of Population Health, University of Oxford, Old Road Campus, Oxford OX27LP, United Kingdom; b Department of Anthropology, University of Amsterdam, Nieuwe Achtergracht 166, 1018WV Amsterdam, Netherlands

**Keywords:** Fieldworkers, Data fabrication, Research integrity, Global health, Ethics, Morals, Morale, Motivation

## Abstract

Data fabrication, incorrect collection strategies and poor data management, are considered detrimental to high-quality scientific research. While poor data management have been occasionally excused, fabrication constitutes a cardinal sin – scientific misconduct. Scholarly examinations of fabrication usually seek to expose and capture its prevalence and, less frequently, its consequences and causes. Most accounts centre on high-income countries, individual senior researchers and scientists who are portrayed as irrational, immoral or deceptive.

We argue that such accounts contain limitations in overlooking data collected in ‘the field’, in low-income countries, by junior researchers and non-scientists. Furthermore, the processes and motivations for fabrication and subversive practices are under-examined. Drawing on two separate ethnographies, conducted in 2004–2009 in medical research projects in sub-Saharan Africa, this paper investigates fabrication among fieldworkers using data from observations and informal conversations, 68 interviews and 7 Focus Group Discussions involving diverse stakeholders. Based on an interpretative approach, we examined fieldworkers' accounts that fabrications were motivated by irreconcilable moral concerns, faltering morale resulting from poor management, and inadequate institutional support. To fieldworkers, data fabrication constituted a ‘tool’ for managing their quotidian challenges. Fabrications ranged from active to passive acts, to subvert, resist and readdress tensions deriving from employment inequalities and challenging socio-economic conditions.

We show that geographical and hierarchical distance between high-ranking research actors and fieldworkers in contemporary configurations of international medical research can compartmentalise, and ultimately undermine, the relationships necessary to produce high-quality data. In focusing on fieldworkers, we argue for the inclusion of wide-ranging perspectives in examinations of data fabrication.

## Highlights

• Unique focus on fieldworker motivations for data fabrication.
• Moves beyond describing fabrications by high-ranking scientists in wealthy contexts.
• Fieldworkers motivations related to moral concerns and morale issues during data collection.
• Argues for focus on institutional factors shaping fieldworker practices.

## 1 Introduction

I am always surprised that people would take that chance [of fabricating data], and yet they [fieldworkers] keep doing it! Maybe you should talk to some of those people and find out what the motive could have been. (Laughter) Because it's true…we always say…why would you take the chance to lose a job, when you know it's so hard to get a job? But it happens. Why go through this process of being one of…hundreds of people to get this job, knowing what the [employment/economic] situation out there is like, knowing the sort of stand that we take about these things – to then falsify the data?

(Eve*, Senior expatriate researcher, In-depth interview, STUDY A)

Data fabrication remains a consistent feature of medical research, and yet, as the senior researcher quoted above points out, its motivations are generally poorly documented. Examinations of data fabrication predominantly derive from high-level researchers and bench scientists in wealthy countries (Mojon-Azzi and Mojon, 2004, Sovacool, 2008). Consequently, our understanding of fabrication barely considers the masses of ‘invisible’ fieldworkers, technicians, students and other ‘hired hands’ involved in quotidian aspects of research work (Roth, 1966, Shapin, 1989, Timmermans and McKay, 2009). Moreover, studies of fabrication rarely involve low-income settings in the global South (Ana et al., 2013, Fanelli, 2009, Okonta and Rossouw, 2014), where a considerable proportion of medical research takes place (Petryna, 2009).

This paper addresses these oversights by examining fieldworkers' motivations in fabricating data in two contemporary Sub-Saharan African research settings. It addresses why, how and when data was fabricated; not the impact of data fabrications on research more broadly. This paper argues that fabrication, from fieldworkers' perspectives, was motivated by moral and morale factors. These factors included: difficulties in reconciling physical, economic and contextual challenges emerging during community-based work with their expected roles; resistance against perceived unrealistic work-loads; discontentment with employment terms and conditions; insufficient institutional support; and management weaknesses.

### 1.1 The inexplicable act: why and by whom is data fabricated?

Data fabrication (inventing data or cases), along with falsification (distorting data or findings) and plagiarism (failure to attribute copied words, ideas or data), are frequently defined as scientific misconduct that share a hallmark feature: the intention to manipulate research outputs (Fanelli, 2009, Franzen et al., 2007). This intentionality is important in distinguishing scientific misconduct from questionable research practices (QRP), which are unintentional deviations, for instance through error or negligence (Steneck, 2006). A recent study reported that almost a quarter of research scientists admitted to intentionally fabricating or falsifying data during their career, while nearly three-quarters engaged in QRP (Ana et al., 2013). Like many other studies, little attention was paid to possible motivations.

Studies on data fabrication in medical research often speculate about its possible motivations. In public and scholarly discussions, data fabrications is commonly framed as an inexplicable act. For instance in April 2013 in the first case of a UK scientist being imprisoned for fabrication, the sentencing judge remarked to Dr. Steven Eaton: “*Why someone…as highly educated and as experienced as you would embark on such a course of conduct is inexplicable*” (BBC News, 2013). The judge's comments point at two further biases in the literature: discussions of fabrications typically focus on acts by high-ranking researchers; the context that shapes the motivations and behaviour of these individuals often gets less attention (Franzen et al., 2007). When contextual issues are discussed, for instance the pressure to publish, how institutions might intentionally or unintentionally foster fabrications is seldom researched in detail (Pryor et al., 2007). Collectively, such biases have skewed accounts of who undertakes fabrication and why it occurs.

### 1.2 Motivations for data fabrication among field-level research actors

Almost seventy years ago, in his account of fabrications among demographers, Crespi (1946) distinguished between *moral* conceptions of fabrication, which framed explanations at individual-level, and *morale*, which had institutional implications. Following Crespi's distinction, data fabrication is not necessarily undertaken by immoral individuals, a common perspective in the medical literature, but can also be undertaken for moral reasons by demoralised employees such as fieldworkers lacking institutional support. Thus, exploring morale can illuminate institutional and social conditions that enable and foster fabrication (de Sardan, 1999).

Sociologist Roth, in an early study investigating the institutional and social conditions underlying data fabrication (1966), traces how ‘hired hands’ shape data collection in various American contexts:

After it became obvious how tedious it was to write down numbers on pieces of paper which didn't even fulfil one's own sense of reality and which did not remind one of the goals of the project, we all in little ways started avoiding our work and cheating on the project. It began innocently enough, but soon boomeranged into a full cheating syndrome, where we would fake observations for some time slot which were never observed on the ward.

[…] Even those who start out with the notion that this is an important piece of work which they must do right will succumb…when they realize that their suggestions and criticisms are ignored…that they will receive no credit for the final product, in short, that they have been hired to do somebody else's dirty work. They will cut corners to save time and energy. They will fake parts of their reporting. They will not put themselves out for something in which they have no stake except in so far as extrinsic pressures force them to.

(Roth, 1966, pp. 190–192)

Despite its focus on an American setting, an enduring value of Roth's work is in foregrounding the consequences of a Fordist knowledge production model (Beynon and Nichols, 2006), which compartmentalises research projects and reproduces a hierarchical division of labour, with negative effects on fieldworker morale. Such compartmentalisation and divisions of labour are now commonplace and permeates countless institutions; biomedical research in sub-Saharan Africa is no exception.

Almost fifty years later, first-hand fieldworker insights are rarely discussed; however, a small but significant body of work, dispersed across numerous disciplines and contexts, has sought to provide contemporary accounts of fieldworker data fabrication. For instance, Biruk (2012) presents valuable ethnographic insights on fieldworker data fabrication in a resource-poor Malawian context, although institutional influences on fieldworker fabrications received limited attention.

A study that specifically examines institutional influences in data fabrication, within a community-based drug-use intervention in a low-income setting in Philadelphia, notes that perceived “procedural injustice in the research enterprise itself… may in turn contribute to misbehaviors in research” (True et al., 2011: 4). With a quarter of participating fieldworkers disclosing fabrication on moral grounds, and half knowing of a colleague who fabricated data, but not disclosing this to superiors, True et al. highlight the relevance of context-specific dynamics and collective action in shaping data fabrication (2011:4–5). This reinforces de Vries et al.'s (2006:44–45) argument that focusing on a single “bad apple” obscures complicity, institutional environments and research contexts that produce or endorse fabrications – a ‘bad barrel.’ Additional work by these authors directly implicates institutional policies and lack of support in fieldworkers' moral distress during data collection (Fisher et al., 2013). While many of these examinations occur in the global North, they challenge ideas that data fabrication is exceptional and underscore the need for investigations of its social and institutional underpinnings.

### 1.3 Genuine fakes

Science and Technology Studies (STS) scholars have long argued scientific data are socially constructed, shaped by wider sociocultural, political and economic forces, and different actors' motivations (Latour et al., 1986). This literature emphasises the inherent contradiction central to objective, decontextualised accounts of data production. To be ‘scientific’ and ‘genuine,’ data must be standardised through controlling for errors and protocol deviations; by contrast, ‘fake’ data is defined as not adhering to scientific methodology (Al-Marzouki et al., 2005). Yet, STS scholars argue that outside of controlled experimental settings such as laboratories, data collection is rarely fully standardised (Lampland and Star, 2009). Rather, uncontrollable variables impinge, challenging the fake/genuine dichotomy regarding data production (Erikson, 2012). A substantial literature concerned with the challenges of obtaining high quality data in African settings underscores the need to investigate the influence of social and institutional contexts on data generation.

While STS examinations typically focus on issues and staff in laboratory, clinical and scientific settings in the global North, their questioning of the binary between ‘fake’ and ‘genuine’ data has wider implications for fieldworkers in community settings. For instance, Waller's (2013) accounts of fieldworkers in Jamaican electoral polling describe some fabrications which yielded factually correct data, called ‘genuine fakes’ (Schraepler and Wagner, 2005, Finn and Ranchhod, 2013). While demographers introduced the notion of curb-stoning – fieldworkers who invent survey data on curbs or pavements without visiting households (Wainer, 2004) – ‘genuine fakes’ involve curb-stoning by fieldworkers, familiar with the location, population and subject-matter, completing surveys without consulting participants, instead using accumulated experience or informed guesswork. Waller's assertion that this type of fabrication subverted institutional procedures and protocols without compromising data quality raises questions for investigations of this phenomenon in African settings. Moreover, since certain problems (such as HIV/AIDS) tend to attract more resources than other issues (Shiffman, 2008), such differences can profoundly influence data quality procedures as evinced by long-standing debates about weaknesses of health data infrastructures in many low-income countries (AbouZahr and Boerma, 2005). The remaining sections of this paper will focus on these issues among medical research fieldworkers.

## 2 Methodological and theoretical approach

### 2.1 Background information about research sites

This paper is informed by two separate ethnographies on fieldworkers, conducted in two different Sub-Saharan African biomedical research institutions between 2004 and 2009. Common themes among these independent ethnographies became apparent after data collection had ended. Multiple methods were employed, including extensive observations, face-to-face interviews, focus group discussions (FGDs) and in one location a graphic elucidation technique. This paper presents data reanalysed by both authors. Presenting these findings together allows for comparing and contrasting cross-cutting themes, while affording increased anonymity for individuals and institutions, important conditions of their participation. To preserve anonymity, locations are renamed as SITE A and B, research institutions INSTITUTION A and B, and the ethnographic examinations STUDY A and B.

#### 2.1.1 SITE A

SITE A was chosen because it employed several hundred fieldworkers in approximately 10 medical research projects and an external researcher was permitted to examine their conduct. Research projects were organised at institutional headquarters and implemented through five field stations and 20 sub-field stations, spanning an area of approximately 20,000 km^2^ with 300,000 inhabitants, and considerable physical and geographical variation. At sub-stations, generally clinics or hospitals, research projects temporarily occupied space for project-related activities.

#### 2.1.2 Fieldworkers

The sub-stations of five projects in STUDY A, where fieldworkers worked, were a three-hour drive from headquarters. Typically, senior scientists determined selection criteria for fieldworkers, then project managers organised recruitment. In many projects, senior scientists and managers preferred fieldworkers from, or near, research communities, to facilitate engagement. Following a short project-related training, fieldworkers would then recruit participants.

#### 2.1.3 SITE B

SITE B was chosen because it hosted anti-malarial drug trial involving a dozen local and international organisations. The leadership had welcomed author2 to conduct an institutional ethnography exploring collaborative practices among stakeholders ranging from senior scientists to clerks and fieldworkers.

Trial-related activities occurred in SITE B, and scientific and administrative activities in INSTITUTION B, a three-hour drive away. SITE B encompassed around 15,000 km^2^ with approximately 200,000 inhabitants, half of whom were monitored by a Demographic Surveillance System (DSS), which hosted research projects investigating infectious diseases and poverty alleviation. INSTITUTION B's rented offices at the DSS, where research activities were organised and implemented, often in cooperation with the district-level health system – a network of two hospitals and about 60 dispensaries.

#### 2.1.4 Fieldworkers

Projects in SITE B often preferred local fieldworkers for their familiarity with communities and hands-on experience. Annually, between 50 and 150 fieldworkers were employed, on contracts of variable length and remuneration reflecting varying per-diems or ‘working away from home’ bonuses. Since declining a job offer might foreclose future opportunities, fieldworkers generally accepted low salaries. As in SITE A, this dependency nurtured an institutional culture which dissuaded raising sensitive topics. This culture influenced the methodological approach taken in both ethnographies (see Table 1).

### 2.2 Background information about ethnographic studies

Both ethnographic studies investigated everyday data collection to understand its contextual influences in large-scale biomedical research operations. Neither study initially examined fabrication but when this topic emerged, it was included.

#### 2.2.1 STUDY A

STUDY A focused on fieldworkers, not on interventions or diseases, although these were considered important influences on design and potential everyday challenges. Research projects were chosen to capture various features and different research staff (including PIs, Study Co-ordinators and Directors) to elicit divergent views.

In STUDY A, examining fieldworkers' daily duties required regular negotiations with Project Co-ordinators and Field Supervisors and to minimise disruption author1 rotated between projects. Fieldworkers were asked to participate in STUDY A in two stages. Firstly, author1 attended each project's weekly meetings, requesting time beforehand to introduce the study. In these meetings, author1 emphasised her independent status and fieldworkers' voluntary participation and distributed information sheets with contact details. Then STUDY A began with observing fieldworkers' day-to-day activities. These findings informed subsequent interviews.

#### 2.2.2 STUDY B

STUDY B ethnographically ‘tracked’ the antimalarial trial by ‘following’ various actors and trial-related activities (Marcus, 1995). Over a two-year period, author2 examined eight trial-related projects, participating in diverse activities such as scientific planning meetings, fieldworker training workshops and routine data collection. During participant-observation in various trial activities, author2 gradually acquainted himself with fieldworkers and their work activities, which formed the basis for subsequent interviewing (see Table 2).

### 2.3 Positionality

Typically, examinations of fieldworkers are conducted by social scientists embedded within (or employed by) the institutions they research (e.g., Kamuya et al., 2013). In this paper, both researchers entered the institutions they studied as outsiders and gradually became “partial insiders” (Sherif, 2001), facilitating certain avenues of exploration while complicating others. The process of becoming partial insiders helped researchers develop contextually appropriate ways of investigating this sensitive topic. In STUDY A, author1 rotated between projects that, generally, involved separate groups of fieldworkers with different senior and junior staff. This cyclical aspect helped establish familiarity and trust with fieldworkers. In STUDY B, author2 traced different projects with considerable staff overlap. Although the leadership supported STUDY B, when author2 began exploring labour issues, several junior researchers withdrew their cooperation. Various fieldworkers took this as a sign that author2 could be trusted, making it possible to broach the sensitive topic of data fabrication, initially during casual conversations and eventually in some interviews.

### 2.4 Methods

Both studies used an iterative data collection strategy to investigate when, why and how fabrication occurred; neither study was designed to quantify fabrication or assess consequences for data quality. Data obtained through participant observation, for instance, demonstrated variations in data collection practices, including violations of procedures. Such findings informed investigation of what informants ‘say they do’, through casual conversations, interviews and focus group discussions (FGDs), informing subsequent participant observation. Complementing dozens of informal conversations, STUDY A involved 31 in-depth interviews and seven FDGs (conducted in English), and STUDY B, 37 interviews (conducted in KiSwahili, translated into English).

Observations demonstrated instances of data fabrication. However, we were unable to verify whether fieldworkers' statements about fabrications pertained to ‘real’ deviations, if or how these affected data. Since in this study fabrications constituted a lens for exploring attending social and institutional issues, it mattered less whether these were ‘real’ or ‘alleged’ (though obviously we recognise that this distinction has different consequences for data quality). Akin to White's investigation of rumours (2000:81–86), we approached talk about fabrication as a meta-commentary on social reality, as a mode of representing and discussing used by informants to raise issues that matter to them.

### 2.5 Theoretical position

Both studies took an interpretative approach to exploring fieldworkers' and other relevant research actors' perspectives, to provide insider and bottom-up insights. This position accepts that respondents perceive, understand and discuss fabrication differently; of interest are the influences on these views. Interpretative approaches and grounded theory influenced data analysis, which combined emic and etic perspectives in inductive investigations. In both studies, interpretations were shared and modified in discussions with informants. This epistemological approach increased transparency in relationships with informants, and contributed to verifying or refuting themes or observations.

### 2.6 Ethical approval

Participants were given study information sheets in English, Kiswahili and local languages and were asked to sign and date a consent form if they wished to participate and be recorded. Participants were given opportunities to ask questions and express concerns. They could refuse to answer questions and/or withdraw from participation at any time. Interview materials were stored securely to assure confidentiality. Ethical approval was obtained from the London School of Hygiene and Tropical Medicine, New York University and all research institutions and national bodies involved.

## 3 Findings

Recalling the quote opening this paper, expatriate researcher Eve* expresses prevalent thinking among senior researchers in SITE A when discussing deviations in data collection and, especially, the presumed frequency of fabrication. Incredulity often permeated their answers; why would fieldworkers fabricate data when fabrications were easily detectable, particularly in survey data? Most senior researchers in SITE A – typically expatriates based at research headquarters with limited in-country contact and field experience – claimed not to understand what motivated fieldworkers to fabricate data. However, in SITE B, senior researchers generally were more attuned to possible motivations for data fabrications, probably because they had greater experience working under field conditions, were usually familiar with local customs and languages, and interacted more frequently with fieldworkers. In both locations, fabrication detection procedures were in place. These varied considerably between projects and depended, for instance, on the type of data being collected, available resources, and management practices. For example, surveys and questionnaires usually contained ‘red herring’ questions: ‘trick’ questions that tested whether fieldworkers had followed guidelines. Supposedly, fieldworkers were unaware of trick questions, and incorrect answers signalled misconduct. The consequences of detected fabrications differed: in SITE A, fieldworkers risked punitive measures, including losing their job whereas in SITE B, they were admonished but fired only after repeat offences.

Aimed mainly at researchers' efforts to obtain reliable data, these quality control procedures revealed little about fieldworkers' motivations for deviating from good practice. Drawing on ethnographic insights, the following sections shed light on these motivations, examining why and how fieldworkers modify data collection protocols to illuminate moral and morale features of data fabrication.

## 4 Moral features of data fabrication

On one occasion, author1 accompanied “Faith,” a 19-year-old fieldworker in SITE A, to visit “Rose,” a study participant interviewed monthly for survey data collection. During a two-hour walk in the scorching heat from the sub-station, Faith explained that Rose, a recently-widowed 23-year-old mother with three children aged under 6, earned, at most, a dollar a day. After exchanging greetings, as Faith commenced the interview, Rose, holding her four-year-old boy, said: “he developed fever last night.” After wishing the boy a speedy recovery, Faith continued interviewing Rose, which took only 25 min instead of the usual 45 min. Faith talked longer than customary with Rose and played with her kids. While saying good-bye, author1 noticed that Faith handed Rose some coins.

Walking to the next household, Faith explained that she shortened the interview when noticing Rose's apparent discomfort with some of the questions. “You mentioned that Rose and her children had lost weight since our last visit,” queried author1 to which Faith replied rhetorically, “So why ask her, ‘When was the last time you had three meals a day?’” Faith explained that asking such seemingly innocuous food-related questions appeared unnecessary and time-consuming, and made her look insensitive because the study she was conducting did not provide food. To navigate this moral dilemma, Faith spared Rose potentially embarrassing questions. Having interviewed her repeatedly, Faith felt confident about Rose's responses. Faith answered the survey questions accordingly.

When author1 returned to this sub-station two months later to accompany Faith on routine data collection, Martin, Faith's field supervisor, explained that she had been dismissed, calling her “unlucky” to be caught “getting data from under a tree”. Apparently, Faith over time had stopped visiting Rose altogether, yet continued submitting completed surveys. This was discovered after Rose's son died shortly before Faith's next scheduled visit, triggering a standard investigation into the circumstances of the study participant's death. Unaware, Faith completed the survey showing that the boy was alive. Researchers detected Faith's fabrication, and fired her. Fully agreeing with this decision, Martin also considered Faith unlucky because she was a ‘good fieldworker’ who cared for her participants but received insufficient support in managing moral challenges that emerged during community-based work. The following sections further examine these themes.

### 4.1 “Data from under a tree” and everyday practical challenges in resource-poor contexts

**Fig. 1:**
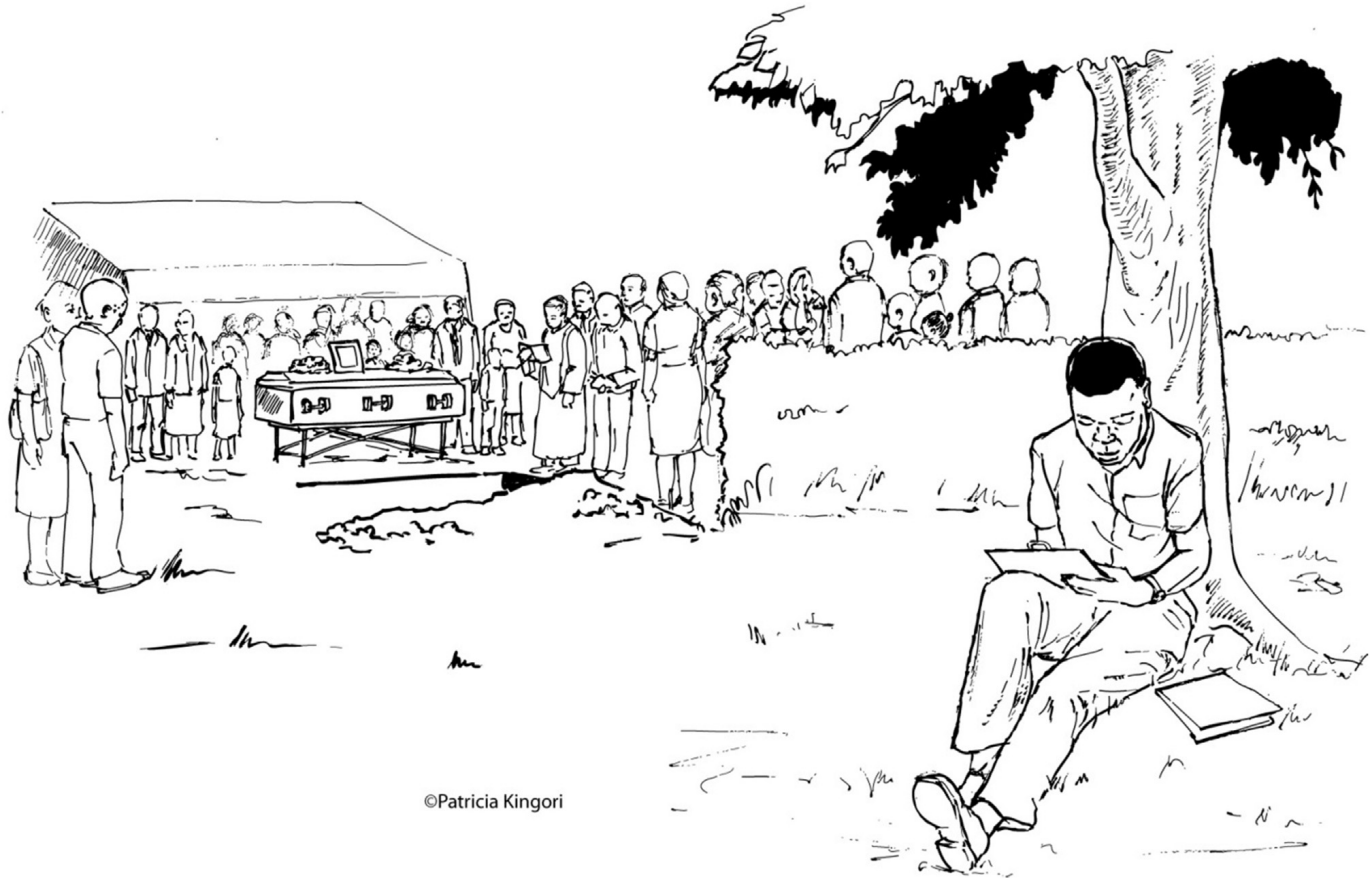
Data from under a tree.

The phrase Martin used – getting “data from under a tree” – surfaced regularly in both sites to indicate real, suspected and alleged data fabrication and research protocol deviations. This euphemism inspired Fig. 1, which portrays commonly encountered predicaments that could motivate fieldworkers to adapt research protocols and fabricate data. Co-produced with fieldworkers in STUDY A through an iterative process of drawing, amendments and analysis, it recalls the situation Faith confronted. In the foreground, a fieldworker sits under a tree, completing a questionnaire but without having collected information from the selected research participant. In the background, relatives and community members are attending a funeral outside the research participant's home.

The funeral depicted in Fig. 1 exemplifies situations that emerge during community-based work and present fieldworkers with dilemmas and pressures. Their efforts to navigate these challenges point at two key issues: fieldworkers' position as intermediaries between communities and the institution they work for, and inadequate institutional support for their community-based activities.

The ‘real-world’ dynamics of community-based activities regularly present fieldworkers with ‘extraneous intrusions’, yet their leeway to accommodate these events often was quite limited. Events such as a sudden death elicited expectations among community members that fieldworkers participate in mourning or contribute to funeral expenses. This could affect recruitment schedules or requiring additional time and funds, but institutional support for addressing such issues was inconsistent or lacking in both sites. Nevertheless, fieldworkers could not ignore community-level wishes or expectations and were compelled to navigate the situations they encountered.

Although both institutions recognised the important role of fieldworkers in developing and sustaining good relations with research participants and the wider community, this was seldom reflected in project design which was rarely sufficiently flexible to accommodate the dynamics of community-based work in contexts of poverty, and deprivation. Manifesting at the level of planning and implementation, this tension is related to research labour. Projects in SITE A generally were designed by PIs with varying knowledge of field conditions, which sometimes resulted in unrealistic planning (an issue elaborated below). Predominantly expatriates, PIs visited the sub-station to supervise the launch of a study and generally returned to project headquarters soon thereafter. Project managers and field supervisors then managed the implementation.

In SITE B, the local and foreign PIs who co-developed a study usually accompanied junior scientists to supervise the launch; then the latter overviewed implementation from a sub-station, returning home for weekends. PIs in both sites mentioned reasons such as their workload, field conditions, or unreliable electricity to explain their limited time at sub-stations or in the field. Especially in SITE A, this stratification of research labour and geographic separation widened the (socio-economic and, on occasion, racial) distance between PIs and fieldworkers, obscuring their working conditions and the issues they faced. In comparison, PIs and junior scientists in SITE B were generally relatively more familiar with fieldworkers and their working conditions. However, clear institutional procedures for addressing such issues were lacking so in practice junior researchers and field supervisors decided what to do. Participant observation showed considerable variation: while some superiors fostered a welcoming environment for raising and resolving work-related challenges, sometimes even covering associated expenditures, others signalled outright hostility. Generally, fieldworkers established quickly which superior was willing or disinclined to help them resolve work-related problems. When support was not forthcoming, fieldworkers tried to solve the problem themselves. In such situations, deviating from research protocols including fabrications could help fieldworkers resolve challenges encountered during community-based work.

Juggling study enrolment criteria is an example of challenges that fieldworkers resolved without consulting superiors when institutional support was lacking. In STUDY A, enrolment criteria were generally determined before a project started, yet during implementation fieldworkers encountered situations that required research protocol modification. For instance, Ferdinand explains why he included an ineligible child in a study:

There was another case where the child…died in her sleep… The mother woke up and found the child was dead. She told me and asked me if her other older child could take her place [of the deceased paediatric participant] to get better care…How could I refuse? I just carried on [collecting data] but seeing this other child…

(Ferdinand, Fieldworker, In-depth interview, STUDY A)

Had Ferdinand enforced the protocol, the death of the eligible child would have meant excluding the family and in turn withdrawing a coveted study benefit: access to better healthcare. Instead, Ferdinand included the deceased child's ineligible sibling, so family members retained their healthcare. Ferdinand's decision violated protocol yet elicited community approval. Demonstrating their support, community members and fieldworkers agreed to keep Ferdinand's protocol deviation secret.

Community approval and collusion in Ferdinand's protocol violation are about more than accessing research benefits. Ferdinand's actions demonstrate to community members that he is a ‘good person’ who shares their moral code. Paradoxically, this shared moral code enhanced community support for the project while undermining its scientific design. This ambiguity attending Ferdinand's actions highlights fieldworkers' ‘moral brokerage’ while engaging in community-based work (Lewis and Mosse, 2006). Juggling whose priorities and morals prevail is merely one example of their brokering.

Observations in both sites revealed that fieldworkers often worried being deemed ‘troublemakers’ when raising work-related moral dilemmas. This reinforced an institutional culture which tacitly assumed that fieldworkers refrain from addressing ethical issues that conflict with scientific goals. True et al. (2011) described similar findings in Philadelphia, despite the radically different social context. They found that community research workers with limited institutional support and oversight subverted protocols by urging ineligible participants to access drug treatment intervention trials to help them improve their lives (see also Timmermans and McKay, 2009). As in Philadelphia, community members in STUDIES A and B appreciated research also for its non-scientific benefits. Fieldworkers realized that modifying protocols could undermine data quality but their primary concern was juggling conflicting social and work pressures stemming from their community-based work. Hence protocol deviations and fabrications could be viewed as a ‘solution’ for managing these practical challenges and moral dilemmas.

Subverting research protocols and fabricating data were not only a means to meet work demands and maintain community cooperation. As will be discussed next, such deviations were also motivated by employment conditions, managerial weakness and poor morale.

## 5 Morale features of data fabrication

Certain research projects, characterised by tight timeframes and pre-set data collection targets, were regularly mentioned as eroding fieldworker morale, the second major justification for subverting research protocols and fabricating data discussed in this paper. Typically, targets are set at the beginning of projects based on diverse criteria such as statistically determined sample size calculations or available funding. The ensuing plan specifying a target number of interviews, questionnaires and participants generally contained limited concessions to contextual factors such as inclement weather. Consequently, rigid planning and everyday dynamics of community-based work were often misaligned. When targets were unmet, this usually generated created stress elsewhere in the plan, for instance, prompting a decision to increase the daily number of interviewees for each fieldworker. This entailed longer working days though sometimes additional pay. Called ‘volunteering’ (KiSwahili – *kujitolea*) in SITE B, such extra work was generally expected. When relations with superiors were good, fieldworkers readily volunteered additional time and effort. However, when these relations were frayed, and superiors demanded that fieldworkers volunteer, dissatisfaction would spread. Eventually, rising dissatisfaction undermined fieldworkers' morale.

Declining morale could progressively impact fieldworkers' work, especially if work plans were inflexible or insufficiently considered working conditions. In both sites, we observed that passive deviations such as corner-cutting could indicate declining morale which, if not remedied, could forebode more active deviations such as fabrications. Fieldworker Faye's declining morale is palpable when explaining why she decided to fabricate data in response to unrealistic collection targets:

…**if you had like 8 interviews to do in a day and those [survey] interviews are quite long distances apart, you're working in the hot sun…that can motivate someone to say, let me just sit under the tree and forget this.** […]I think in **the process of doing data collection, at times there is a way in which you can predict trends.** Once you've been doing data collection…if you've visited him for the last three months […] **you could predict the flow of information…you are getting from a particular client.** So if you're having a lot of work on a particular day, you would think, ‘For this one I know, this is the pattern on how she's been answering these questions', then you can just sit down and answer that.

(Faye, Fieldworker, In-depth interview, STUDY A – Emphasis added)

In situations as described above, fabricating data can become a ‘solution’ to pressures stemming from tight project planning or challenging working conditions. Drawing on her accumulated knowledge about participants and their living conditions, Faye decided to collect ‘genuine fakes’ – data she considered accurate but not collected according to protocol. Ongoing familiarity with research participants' lives facilitated collection of genuine fakes but did not necessarily protect against remaining undetected, as Ferdinand's case and Faith's dismissal demonstrated. Hence, fieldworkers generally viewed generating genuine fakes as a short-term strategy for solving acute problems.

Fieldworkers disclosed that protocol deviations extended beyond questionnaires or surveys, and involved other kinds of data such as stool or blood samples. Observations in both sites confirmed these claims. For instance, Frank explained that he substituted blood samples when having insufficient time or funds during data collection. He explains that:

Maybe the distance to the clients' home is far…the road is poor…and you don't have money to help them. Therefore…I might not go there. I might even bleed my kid or another relative.

(Frank, Fieldworker, In-depth interview, STUDY A)

Collecting specimens from non-participants occurred in both sites, for various reasons. Some fieldworkers collected extra samples to compensate for non-compliant or absent participants, without recording this deviation. They reasoned that if it looked like they adhered to protocol and no suspicion was aroused, the substitution might never be discovered, or well after data collection had ended, diminishing chances of identifying who fabricated data. A statistician in SITE B confirmed this assessment, and explained that the ability and need to detect fabrications varied considerably between projects, due to variables such available resources and staff, the kind of data collected, and perceived urgency in relation to quality control requirements.

### 5.1 “Good and bad supervisors”

As mentioned earlier, project managers or junior researchers were responsible for running projects day-to-day. Observations in both sites showed that this division of labour created ‘implementation managers’ who displayed diverse styles of managing projects and interacting with subordinate staff, aspects that could influence fieldworkers' morale for better or worse. Although official policies governing staff conduct existed in both institutions, the decisions and interpretations of powerful managers often superseded these. If scientific goals were met and project accountants certified expenditures, PIs or other senior staff seldom intervened in day-to-day management. Therefore, implementation managers managed as they saw fit and held considerable power over projects and staff. These structural power differences form the backdrop to examine data fabrications as a reaction against poor management practices that undermine fieldworker morale.

That management practices can influence staff morale motivate data fabrication is illustrated in the following example involving John, an experienced fieldworker who recounted a country-wide sanitation and health study. INSTITUTION B tasked a junior researcher, Bakari, with managing the study in SITE B and adjacent districts. Subsequently, Bakari initiated a routine procedure: he asked local gatekeepers to pre-select about twenty candidates for a workshop-based training, during which twelve candidates would be selected and employed on short-term contracts. John was selected, and the following day, two Toyota Landcruisers transported the team – Bakari, a field supervisor and six pairs of fieldworkers – to an adjacent district, dropping each fieldworker pair at pre-selected locations. John and Mohamed, also from SITE B, were dropped in Mtupa, a market town.

After disembarking, Bakari gave John and Mohamed final instructions and about US$45 each – advance wages for their weekly lodging and food. After settling into a guesthouse, they began planning their work. At 40 pre-selected households in and around Mtupa, they were supposed to interview each head and all resident women with children under five, collect stool and blood samples from these children, and observe sanitary facilities and homestead conditions. When John and Muhamed realized that these households lay scattered across a large area, some well outside town, they pondered how to accomplish their work without transport funds. Answering the survey containing nearly 100 questions could take approximately three hours, if done diligently. Completing 8 households daily was impossible without transport. Moreover, it was cultivation season; locating participants at outlying farms could take substantial time and effort. To resolve this challenge, John and Muhamed modified the project protocol: they enlisted a local government official to direct participants to a nearby location. There they interviewed, sampled and weighed them, using what John called the ‘rapid method’:

I come here [Mtupa] to weigh and test little children…and collect those forty mothers. Thus you compress a week of work into a day. You list everyone's names, test them and fill out everything at the guesthouse. Then you move on. Otherwise you lose time, because they [supervisors] were greedy. If you'd work as required, it would take longer and you'd have insufficient money to pay for your room and food.

Therefore you have to adopt the rapid method– *mbinu wa haraka* – and finish the job in one day. And then you leave…**If people face difficulties with money, they also think about what kind of data they want to give you.**

(John, Fieldworker, In-depth interview, STUDY B Emphasis added)

John's justification for modifying the protocol by the ‘rapid method’ links two key points: perceived unfair employment conditions, and poor management practices – exemplified by ‘greedy’ supervisors, thereby undermining proper protocol implementation. The superior could have forgotten to issue travel funds, but John insinuated that these had been pocketed. Although policies penalizing financial improprieties existed in SITE B, implementation was uneven. Supervisors intent on misappropriating oftentimes succeeded, spurring talk and rumours about ‘greedy bosses’ who ‘ate funds,’ and fuelling a culture of impunity, suspicion and distrust that eroded morale (see also de Sardan, 1999).

Echoing findings presented above, John expressed little concern that supervisors would detect that he modified the study protocol. Having worked over a decade as fieldworker or supervisor in and beyond SITE B, he counted on his experience to recognise what kind of genuine fakes should be collected to avoid getting caught:

…so if supervisors despise data collectors, we are … collecting the data. **If we want to damage the data, nobody will know if this is true data or not.**

They will accept it as is[…]so you [supervisor] are thinking, this person [fieldworker] is collecting blood slides and [you] failed to pay him on time. The person then looks at the environment thinking, there is not much malaria here, and pricks himself for blood. **He collects many samples [from himself] and writes down different names, but the blood is all his. Nobody will ever know this**.

(John, Fieldworker, In-depth interview, STUDY B – Emphasis added)

That management issues can influence fabrication has been known since Roth's ground-breaking study (1966), but to understand how these issues feature in sub-Saharan settings it is important to consider local labour practices. In both sites, scarcity of well-paid jobs and widespread un(der)employment meant that getting and keeping jobs presented fieldworkers with various challenges. Following national law, vacancies were advertised publicly in both sites. However, in practice, as with the nutrition study above, in SITE B some fieldworkers were recruited through ‘connections’ (e.g., relatives) and most through gatekeepers controlling informal labour reservoirs of readily available, skilled candidates. Overall, hiring patterns reflected the central role of well-positioned ‘patrons’ who reinforced their power by dispensing scarce jobs and other favours to ‘clients’ (de Sardan, 1999). As one fieldworker explained: *“if there is nobody to help you, you are unable to get a job!”*

Observations showed that fieldworkers in SITE B generally valued this dependency when patrons and other superiors treated them ‘fairly’, a notion that encompassed adequate pay, respectful treatment, and willingness to discuss work-related problems. When so-called ‘good bosses’ treated subordinates fairly, a cooperative spirit flourished; fieldworkers usually worked diligently and observed protocol. However, perceived unfair treatment or abuse from ‘bad bosses,’ produced poor morale and deviations from protocols increased.

Biruk described the need to implement research protocols as intended, notably the requirement in survey research that fieldworkers “ask every question” (2012:358) as a source of friction between fieldworkers and community members concerned about the lengthy nature of meticulous questioning. Similar ideas were found in our studies, but observational and reported data identified other prominent sources of friction: institutional policies, managerial practices and perceived maltreatment by superiors. Notwithstanding their lowly position in institutional hierarchies and considerable dependence on superiors, fieldworkers reacted to unfavourable working conditions and ‘bad bosses.’ Fearing negative consequences, most became increasingly reticent, avoided raising work-related problems, and pretended to work normally. Yet as Juma explains below, as morale and motivation faltered, willingness to probe for answers to survey questions declined:

…if I ask…the direct question…in the questionnaire and he does not give the answers, I can twist the questions to make sure that he understands and…gives…the answers I want. But if I have poor relations with the managers or with the supervisors…I will just want to get rid of the activity…in such a way to make it look like I did it

(Juma, Fieldworker, in-depth interview, STUDY B)

Above Juma describes omissions – not ascertaining full comprehension of survey questions, not probing for clearer or substantial answers – that can be seen as one end of a spectrum, passive deviations from research protocols. At the other end of the spectrum are active, deliberate transgressions such as swapping and substituting stool and blood samples or inventing answers.

### 5.2 Fieldworker science

Fieldworkers who discussed deviations and fabrications sometimes evinced a certain pride in their ability to observe and predict trends in data they collected, yet researchers in both sites advocated adherence to scientific models and procedures. Reflecting this attitude, Faye, quoted above, did not discuss her actions using pejorative terms such as *“fabrication”* or *“data cooking”*; rather, she used scientific terms to highlight that long-term observations and inference helped her to *“predict the flow of information”* and *“patterns”* in participants' responses. With similar experience-based confidence, John and Muhamed described their *“rapid method”* to complete questionnaires; accumulated experience enabled them to alter data collection with perceived negligible risk of detection. Some experienced fieldworkers, such as John, prided themselves in spotting ‘red herring’ questions; less experienced fieldworkers reported less risky strategies such as collecting blood samples from non-trial individuals, which they considered harder to detect.

While fieldworkers expressed pride in their ability to assess data trends, most also scorned fabrication although not always because it harms scientific aims but because it could jeopardise their current and future employment opportunities and social connection, all greatly valued in contexts of mass un(der)employment. Since an employed person usually provides financial support to extended family, relatives can exert overwhelming pressure to retain employment at any cost. However, fieldworkers were sometimes faced with circumstances which made them consider altering research protocols and fabricating data to honour this commitment.

## 6 Discussion

In seeking to understand fieldworker motivations for data fabrication rather than merely uncovering or describing its occurrence, this paper has examined their particular perspectives on these acts. Fieldworkers' motivations for fabrication emphasised underlying moral and social reasons and pointed to institutional issues. Fieldworkers adjusted study protocols in response to moral dilemmas occurring during community-based work in resource-poor contexts. Others fabricated data because superiors' actions undermined their morale. Following Scott (1985), fieldworkers' deviations and fabrications can be interpreted as *“a weapon of the weak”* which in some ways empowered fieldworkers to navigate their moral and morale challenges presented during their work. This concept emphasises fieldworkers' agency, expressed diversely in our findings. Although fieldworkers generally occupy low-level positions with little influence within institutional hierarchies, their protocol deviations, which ranged from passive to active, illustrate their influence over data quality during its collection.

The findings of the two ethnographies highlighted that fieldworkers often conceived of themselves as arbiters between ‘genuine’ and ‘fake’ data, with the power to determine which type of data was submitted to superiors. This power can be used by collecting ‘good’ data in keeping with study protocols but also against institutions that inadequately reward or exploit them, or against perceived unfair or abusive *‘bad bosses’* by fabricating data. Viewing fabrication as a manifestation of agency underscores another role of fieldworkers: as “key brokers” in mediating interactions between projects and communities, they can shape study implementation in obvious and subtle ways (Lewis and Mosse, 2006:1–2).

While protocol deviations and fabrication can be viewed as expressing fieldworkers' agency, these practices did little to alter underlying macro-structural conditions influencing research that prompted their actions. Fabricating data rarely improved weaknesses in institutional management, inflexible planning, unsatisfactory employment conditions or the structural inequalities which are commonly documented in research contexts in the global South (Singer and Clair, 2003). Furthermore, producing genuine fakes appeared to be dependent on numerous factors including research design and structure of research projects, or collusion between fieldworkers and community members. Moreover, while fieldworkers felt that they had the power to determine data quality without detection this view was not supported by certain acts of fabrication regularly being uncovered during institutional investigations. Rather, when fabrication was discovered, fieldworkers risked penalisation; the supervisor who fostered conditions that rendered such acts a plausible option for fieldworkers was rarely held accountable. Despite these features fieldworkers fabricated data. As institutions seldom probed fieldworker motivations, their underlying reasons remained unknown or strategically ignored (McGoey, 2012). However, ignoring or denying the complexities involved in data collection was cited by fieldworkers as further motivating their acts of fabrications.

The ability to ignore, deny or remain unaware of fieldworkers' experiences and challenges is assisted by the contemporary configuration of research which favours the compartmentalisation of tasks and personnel by hierarchy, division of labour and distance – e.g., geographic, socio-economic – that we observed during our studies. From fieldworkers' perspectives, these conditions resulted in limited contact with institutional headquarters and scarce involvement in data analysis or producing publications; conversely, those designing projects and conducting data analysis often had limited insight into everyday fieldworker challenges, having delegated these features to project managers. Yet, it was precisely this limited institutional oversight which further buttressed fieldworkers' sense of autonomy, and allowed scope for deviations from unrealistic and challenging protocols and reduced the detection of fabrications.

It was under these conditions that fieldworkers felt confident that they were producing ‘genuine fakes’ – data that appears correct – by drawing on their accumulated knowledge. Although this study was not designed to investigate the prevalence of genuine fakes or their impact on data, our findings questions the extent to which such practices form part of fieldworkers' strategies against moral and morale challenges during everyday data collection. While successfully predicting trends and subverting research protocol afforded fieldworkers a certain amount of pride in their ability and a sense of developing their own ‘fieldworker science’ which stood in contrast to their lowly positions, this paper raises important questions about whether fieldworkers should have to devise these strategies and whether institutions and senior researchers should take greater responsibility and provide more support to fieldworkers.

## 7 Conclusion

Research actors fabricate data for numerous reasons. From fieldworkers' perspective, fabrication was generally motivated by moral concerns and poor morale during routine work. These motivations not only varied greatly from those presented among senior researchers, but fieldworkers' fabrications also involved different strategies such as collusion and producing ‘genuine fakes’. These forms of fabrication draw attention to the challenges involved in everyday data collection in low-income contexts, the moral economies of research and how institutional policies and practices towards fieldworkers shape data quality. The findings presented in this paper show that the geographical and hierarchical distance often found between senior researcher and fieldworkers serve to undermine the relationships necessary to produce high-quality data and for fieldworkers' motivations for fabrications to be understood. While the findings presented and discussed in this paper were collected in Sub-Saharan African contexts, they are relevant to research conducted in a number of different locations, particularly where highly-stratified and compartmentalised research is conducted against a background of social and economic inequality, high unemployment and poor labour laws.

## Figures and Tables

**Table 1:** Key similarities and differences between SITE A and B.

Similarities	Differences
Research institutions with multiple international collaborations between African research centres and European/American organisations and funders	Adjacent countries
Highly stratified division of labour: - Senior-level European/North Americans: grant applications, research design, conference presentations and publication writing - Junior-level African scientists/managers: daily management drafting publications - African fieldworkers: data collection	Nationality of senior researchers: SITE A: European and North Americans hold most senior positions SITE B: African senior scientists co-directed projects with European/North American counterparts.
Most fieldworkers recruited from close to institutions, or through ‘gatekeepers’ to informal labour reservoirs.	Research projects examined: SITE A – 5 SITE B – 8
Fieldworkers' routine activities at considerable distances from headquarters and senior researchers' base. Oversight of fieldworker activities varied but consisted mostly of occasional unannounced visits	Employment contracts: SITE A: Generally under a year SITE B: Usually several weeks or months
Type of research projects examined: - RCTs (involving e.g., malaria and HIV/AIDS) - Survey research (tracking e.g., nutritional status) - Demographic Surveillance System (DSS)	Principal language at research institution: SITE A – English SITE B - Kiswahili

**Table 2:** Key features of fieldworkers participating in STUDY A and B.

Features	STUDY A	STUDY B
Education level	Minimum of secondary school level qualifications	Ranging from secondary school level qualifications to advanced university degree (Masters)
Employment history	Included experience in public sector positions e.g. nursing and teaching. Former employees of NGOs	As in SITE A. Some fieldworkers engaged in farming or day labour
Age	18–35 years	18–41 years
Languages spoken	All fieldworkers spoke the national language, at least one local language and English	All fieldworkers spoke the national language KiSwahili. Highly educated and many experienced fieldworkers spoke English
